# Reliability and validity of the Physical Activity Scale for the Elderly (PASE) in patients with hip osteoarthritis

**DOI:** 10.1186/1471-2474-13-26

**Published:** 2012-02-21

**Authors:** Ida Svege, Elin Kolle, May Arna Risberg

**Affiliations:** 1 Norwegian Research Center for Active Rehabilitation, Department of Orthopedics, Oslo University Hospital, Ullevaal and Hjelp 24 NIMI, Oslo, Norway; 2 Department of Sports Medicine, Norwegian School of Sport Sciences, Oslo, Norway

## Abstract

**Background:**

Physical activity (PA) is beneficial in reducing pain and improving function in lower limb osteoarthritis (OA), and is recommended as a first line treatment. Self-administered questionnaires are used to assess PA, but knowledge about reliability and validity of these PA questionnaires are limited, in particular for patients with OA. The purpose of this study was to evaluate the reliability and validity of the Physical Activity Scale for the Elderly (PASE) in patients with hip OA.

**Methods:**

Forty patients with hip OA (20 men and 20 women, mean age 61.3 ± 10 years) were included. For test-retest reliability PASE was administered twice with a mean time between tests of 9 ± 4 days. Intraclass correlation coefficient (ICC), standard error of measurement (SEM) and minimal detectable change (MDC) were calculated for the total score and for the particular items assessing different PA intensity levels. In addition a Bland-Altman analysis for the total PASE score was performed. Construct validity was evaluated by comparing the PASE results with the Actigraph GT1M accelerometer and the International Physical Activity Questionnaire (IPAQ), using the Spearman rank correlation coefficient.

**Results:**

ICC for the total PASE score was 0.78, with relatively large error of measurement; SEM = 31 and MDC = 87. ICC for the intensity items was 0.20 for moderate PA intensity, 0.46 for light PA intensity and to 0.68 for vigorous PA intensity. The Spearman rank correlation coefficient between the Actigraph GT1M total counts per minute and the total PASE score was 0.30 (*p *= 0.089), and ranging from 0.20-0.38 for the different PA intensity categories. The Spearman rank correlation between IPAQ and PASE was 0.61 (*p *= 0.001) for the total scores.

**Conclusions:**

In patients with hip OA the test-retest reliability of the total PASE score was moderate, with acceptable ICC, but with large measurement errors. The construct validity of the PASE was poor when compared to the Actigraph GT1M accelerometer. Test-retest reliability and construct validity revealed that the PASE was unable to assess PA intensity levels. PASE is not recommended as a valid tool to examine PA level for patients with hip OA.

## Background

Physical inactivity is considered to be a risk factor for many life-threatening diseases and regarded as a major burden on general public health, therefore international and national guidelines recommend that all adults engage in moderate to vigorous physical activity (MVPA) for at least 30 minutes per day[1-3]. Patients with OA are found to be less physically active than the general adult population, and fewer fulfill the recommendations of 30 minutes MVPA per day[4,5]. Being physically active according to the recommended guidelines is beneficial in preserving function and reduce symptoms[6], and PA is recommended as a first line treatment that should be offered to all individuals with hip or knee OA[7,8]. The efficacy and importance of PA and exercise for patients with OA of the lower limbs have been emphasized in several studies[9-12].

Valid and reliable methods for PA assessment are essential for studying its health effects. Frequency, duration and intensity are important factors when evaluating PA as a protective factor against OA progression and functional decline[13]. Numerous methods for assessing PA are available, and can be categorized into three main groups; self-reported assessments (questionnaires, rating scales, diaries), activity monitors (accelerometers, pedometers, heart rate monitors) and direct assessment of energy expenditure (doubly labelled water, indirect calorimetry). Self-administered questionnaires, including the Physical Activity Scale for the Elderly (PASE), can potentially capture all types of activities and allow grading by intensity. They are widely used, due to being inexpensive and easy to administer, and are considered particularly useful in large epidemiological and longitudinal studies. However, questionnaires have obvious weaknesses considering recall and reporting bias. In contrast, accelerometers offer a method for measuring body acceleration, and thereby quantify amount and intensity of movement[14]. Accelerometers often serve as a comparator when validity of questionnaires is evaluated, as they are expected to measure the same construct[15].

Despite the fact that many self-administered questionnaires are available, evidence for validity and reliability is limited [13]. PASE has been found to significantly correlate in expected directions with physical performance, knee pain and knee functioning in patients with knee pain[6,16], and previous studies have reported correlation coefficients of 0.16, 0.43 and 0.49 when compared to an accelerometer in the general, elderly population[17-19]. However, the validity of PASE has not been evaluated in patients with hip OA by comparing it to an accelerometer. The purpose of this study was therefore to evaluate the construct validity and the test-retest reliability of the Norwegian version of the Physical Activity Scale for the Elderly (PASE) in patients with hip OA.

## Methods

### Subjects

Forty patients with hip OA from a larger ongoing randomized controlled trial (RCT), evaluating the effect of patient education and supervised exercise in patients with hip OA[20], were included. Inclusion criteria were age between 40 and 80 years, uni- or bilateral hip pain for more than three months, Harris Hip Score[21] between 60 and 95, and radiographically verified hip OA according to Danielsson's criteria[22]. Patients with low back pain or knee pain, trauma or functional impairments, or diseases that might interfere with participation were excluded. Patients who had gone through total hip replacement surgery (THR) since inclusion in the RCT were also excluded. During September 2010, 61 patients who had been included in the original RCT between 2006 and 2008, were re-contacted and requested to participate in this validation study. Twelve patients did not respond, eight had gone through THR surgery and one lived abroad. The remaining 40 patients agreed to participation and were included in the study.

Anthropometrical (age, gender, height, weight) and sociodemographic data (work status, educational level), as well as data on Harris Hip Score, minimal joint space width, bilateral hip pain and pain duration was recorded at time of inclusion in the original RCT. Data on age has been altered to reflect the actual age at the time of data collection in this validation study.

The study was approved by The Regional Committee for Medical Research Ethics for South-Eastern Norway. All participants received both oral and written information and signed a written informed consent, before inclusion. The data collection was carried out in accordance with the directives given in the Declaration of Helsinki.

### Outcome measurements

PASE is a brief, self-administered, 7-day recall questionnaire designed to assess PA in older adults[23]. It has also been used in studies assessing PA in patients with OA[24,25]. In this study we used the Norwegian version of the PASE, which was slightly adapted when translated due to cultural differences[26], i.e. the question in the original version addressing walking activities was incorporated in the three questions addressing light, moderate and vigorous PA activity. It consists of 24 questions in total and the overall PASE score ranges from 0-315 (and above). The instructions for use and scoring given in the PASE Administration and Scoring Manual were followed (http://www.neri.org). The questions included in PASE address leisure-time, household and work-related PA, with the different items weighted differently. Participation in leisure-time PA, including light, moderate and vigorous PA intensity, and strengthening activities, is recorded as never, seldom (1-2 days per week), sometimes (3-4 days per week), and often (5-7 days per week). Duration is categorized as less than 1 hour, 1-2 hours, 2-4 hours and more than 4 hours. Housework activities are recorded as yes or no, and paid or unpaid work, requiring some PA, is recorded in hours/week. The total PASE score is computed by multiplying time spent in each activity (hours per day) (for leisure and work-related activities) or participation (yes/no) in an activity (for household-related activities), by empirically derived weighting, and then summarizing all items[26]. From the PASE recordings we calculated the total PASE score, representing the overall activity level. In addition we calculated the PASE score for household-/work-related activities and the PASE score for leisure-time PA, as well as the PASE score from the items addressing light, moderate and vigorous PA intensity.

Construct validity of the PASE was evaluated by comparing it to the Actigraph GT1M accelerometer (ActiGraph, LLC, Pensacola, FL, USA) and to the short form of the International Physical Activity Questionnaire (IPAQ). The Actigraph GT1M is an electronic motion sensor comprising a single plane (vertical) accelerometer. Movement in the vertical plane is detected as a combined function of the frequency and intensity of the movement. Counts are summed over 10 second epochs and downloaded to memory. All sequences of 60 minutes or more of consecutive zero counts were excluded from each individuals recording. For the analyses, a valid day was defined as having 10 or more hours of monitor wear. Six or more valid days of registration were considered sufficient. Accelerometers were initialized and downloaded using the software program ActiLife (ActiGraph, LLC, Pensacola, FL, US). Data were reduced using the SAS-based software program (SAS Institute Inc., Cary, North Carolina, USA) called CSA Analyzer (csa.svenssonsport.dk). From the Actigraph GT1M registrations we calculated average counts per minute representing the overall activity level. In addition we calculated total minutes spent in 0-99 counts per minute, 100-2019 counts per minute, 2020-5999 counts per minute and above 6000 counts per minute, representing minutes spent inactive, and in light, moderate and vigorous PA intensity, respectively[27,28]. The proportion of patients who achieved the recommended 30 minutes of daily MVPA was established by dividing total time in MVPA by the number of valid days of recording, giving an average (minutes per day) across the assessment period.

The development of the IPAQ was initiated in 1996, and conducted by an International Consensus Group, with the intention to develop a measure suitable for assessing population levels of PA across countries[29]. IPAQ is a short, self-administered, 7-day recall questionnaire designed for assessing PA in adults. It consists of seven questions which include PA in all contexts of everyday life, and addresses days, hours and minutes spent on vigorous PA, moderate PA and walking. A question on sitting hours per day is also included. The IPAQ is scored by using the Metabolic Equivalent of Task (MET) method, where different activities and levels of intensity are given different MET estimates. In this study the Norwegian version of the IPAQ short form was used, as well as instructions given in the IPAQ Scoring Protocol, both described at http://www.ipaq.ki.se. For the IPAQ we calculated the total MET-minutes per week, representing the overall activity level. In addition we calculated MET-minutes per week for walking activities, moderate activities and vigorous activities.

### Procedures

**Figure 1:**
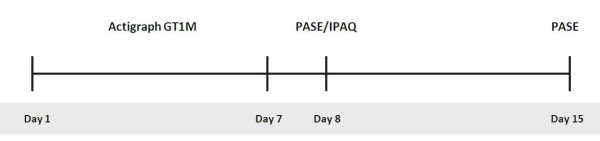
**Schematic view of the timeline of the study**. *PASE*: Physical Activity Scale for the Elderly; *IPAQ*: International Physical Activity Questionnaire.

Data collection for the evaluation of test-retest reliability and construct validity was carried out during October 2010. The Actigraph GT1M was administered by postal mail to all included patients, and it was worn in an elastic belt placed on the right hip. All participants were instructed to wear the accelerometer during all waking hours, except during bathing and swimming, over a period of seven consecutive days (1^th ^-7^th ^day), se Figure 1. The questionnaires, PASE and IPAQ, was administered to the participants by mail on the 7^th ^day, and filled in on the 8^th ^day, the day after finishing the accelerometry registration period, and returned by mail. For evaluation of test-retest reliability PASE was also filled out seven days later (on the 15^th ^day).

### Analysis

Baseline characteristics and descriptive data for the Actigraph GT1M, the PASE and the IPAQ calculations are presented as mean and standard deviation (SD) or number and percentage (%). To evaluate the test-retest reliability for the total PASE score the intraclass correlation coefficient (ICC_2.1 _- two-way random effect model, absolute agreement) was calculated. In addition, ICC_2.1 _was calculated for the sub-score for household/work-related PA, the sub-score for leisure-time PA, and for the PASE score of the items for light, moderate and vigorous PA intensity. Measurement error was assessed by estimating the standard error of measurement (SEM), minimal detectable change (MDC) and limits of agreement (LoA). SEM was calculated as the square root of the within-subject total variance of an ANOVA analysis, *SEM *= √*var_tot_*, and the MDC was calculated as *MDC *= 1.96 × √2 × *SEM *[30]. LoA were calculated according to the Bland-Altman method and a Bland Altman plot for visual judgment of the relationship between the individual mean total PASE score of the test and retest, and the difference in total PASE score between test and retest was made[31].

The construct validity of the PASE was evaluated by calculating the Spearmans rank correlation coefficients (*ρ*) for the total PASE score and the Actigraph GT1M (total counts per minute), and for the total PASE score and the total IPAQ score (total MET-minutes per week). A priori hypotheses were made based on previous studies comparing PA questionnaires and PA measured by accelerometry. As recommended by Terwee et al.[15], the most similar constructs of the PASE and the Actigraph GT1M were compared. We hypothesized a low to moderate positive correlation (*ρ *between 0.15 and 0.5) between the total PASE score and the Actigraph GT1M counts per min. We hypothesized a moderate to strong positive correlation (*ρ *between 0.6 and 0.9) between the total PASE score and the IPAQ total MET-minutes per week. Terwee et al.[15] suggested that the correlation between a PA questionnaire (total score) and accelerometry (counts per minute) should exceed 0.5. We therefore interpreted this as a cut-off for acceptable validity.

In addition, Spearmans *ρ *were calculated for the PASE items for light, moderate and vigorous PA intensity and the different intensity levels/categories assessed by the Actigraph GT1M and IPAQ. For these comparisons the approach was more explorative, but the PASE score for the different intensity items were hypothesized to correlate most strongly with the respective categories of the Actigraph GT1M and the IPAQ as follows: 1) the PASE light PA intensity with the Actigraph GT1M minutes of light PA intensity and the IPAQ walking MET-minutes per week, 2) the PASE moderate PA intensity with the Actigraph GT1M minutes of moderate PA intensity and the IPAQ walking MET-minutes per week and IPAQ moderate MET-minutes per week, and 3) the PASE vigorous PA intensity with the Actigraph GT1M minutes of vigorous PA intensity and the IPAQ vigorous MET-minutes per week.

All statistical analyses were performed using the PASW Statistics 18 for Windows (IBM Corporation, Route, Somers, NY, USA).

## Results

All 40 patients completed PASE at day 8, but at day 15 PASE were missing or inadequately filled out for seven patients. Calculation of the test-retest reliability was therefore based on the 33 patients with complete PASE questionnaires both at test and retest. Thirty-six patients had completed the Actigraph GT1M recording period and had readable files. Two patients returned the Actigraph GT1M unused, and data from two patients were not successfully downloaded. Six or more days of registration were considered to be sufficient. Three patients had less than six days of registration and were thus excluded from the analysis. In total, recordings from 33 patients were included to calculate correlation coefficients between the PASE and the Actigraph GT1M. The average days of registration were 7.0 (0.6). For the IPAQ, 15 patients had missing or incomplete questionnaires, leaving 25 patients to be included to calculate correlation coefficients between the PASE and the IPAQ. This was mainly due to inability to calculate the IPAQ score because the response alternative "don't know" was chosen.

Demographic and clinical characteristics of the patients are shown in Table 1. Based on the Actigraph GT1M measurements 67% fulfilled the recommendations of at least 30 minutes of accumulated MVPA per day, and 30% fulfilled the recommendations of at least 30 minutes of MVPA per day in blocks of minimum 10 minutes. At average the patients spent 45 (32) minutes per day on MVPA.

**Table 1:** Demographics and clinical characteristics of the 40 patients

Variables	
Age in years, mean (SD)	61.3 (10.0)

Men, n (%)	20 (50)

Body mass index, kg/m^2^, mean (SD)	24.5 (3.6)

Years of education, n (%)	

7-9 years	11 (28.2)

10-12 years	13 (33.3)

> 12 years	15 (38.5)

Work status, n (%)	

At work	26 (66.7%)

Retired	10 (25.6%)

Sick-leave	3 (7.8%)

Bilateral hip-pain, n (%)	30 (75)

Minimal joint space in most painful hip, mm, mean (SD)	2.5 (0.9)

Pain duration, months, mean (SD)	49.8 (55.4)

Harris Hip Score, mean (SD)	80.7 (7.9)

### Test-retest reliability

**Figure 2:**
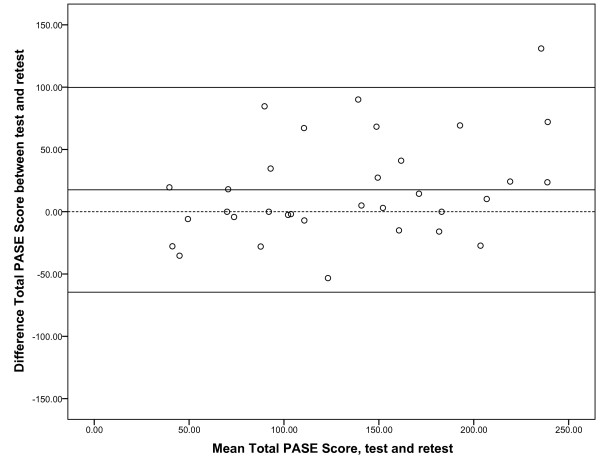
**Bland-Altman plot for total PASE score**. Intra-individual differences (n = 33) plotted against the difference between test and retest scores for the total PASE score. The central horizontal line represents the mean difference, while the flanking lines represent the 95% limits of agreement. The dotted line represents no difference between test and retest.

Mean days between test and retest was nine days (SD 4.0), ranging from six to 25 days. Mean PASE score at test (n = 33) was 143 (SD 71) and at retest 125 (SD 56). The decline in the total PASE score from test to retest was significant (*p *= 0.02), but no significant differences was revealed for any of the sub scores/items. ICC_2.1 _for the total PASE score was 0.77, SEM was 31 and MDC was 87 (Table 2). Test-retest values for the different sub scores/items are also shown in Table 2. The Bland Altman plot for the total PASE score is shown in Figure 2. The lower LoA was -65 and the upper LoA was 100. One out of 33 values (3%) was outside the LoA.

**Table 2:** Test-retest reliability of the PASE

PASE score	Test, mean (SD)	Retest, mean (SD)	*d*, mean (95% CI)	ICC_2.1 _(95% CI)	SEM	MDC
Total score (n = 33)	143 (71)	125 (56)	18 (-3,-32)	0.77 (0.56, 0.88)	31	87

Household/Work activities	114 (63)	84 (59)	15 (-1, 30)	0.69 (0.46, 0.84)	32	89

*Leisure time *PA	29 (24)	26 (19)	3 (-4, 10)	0.53 (0.24, 0.74)	15	40

Light PA intensity	13 (22)	10 (11)	3 (-4, 9)	0.46 (0.15, 0.69)	13	35

Moderate PA intensity	9 (10)	9 (13)	1 (-5, 6)	0.20 (-0.16, 0.51)	10	28

Vigorous PA intensity	4 (6)	5 (7)	-1 (-2, 1)	0.68 (0.44, 0.83	4	10

*PA *physical activity, *PASE *Physical Activity Scale for the Elderly, *d*: difference between test and retest, *ICC_2.1 _*intraclass correlation coefficient, two-way random effects ANOVA, *SEM *standard error of measurement, *MDC *minimal detectable change

### Construct validity

The Spearman's rank correlation coefficient (*ρ*) between the PASE score and the Actigraph GT1M, and the PASE score and the IPAQ score is shown in Table 3. The correlation between the total PASE score and the Actigraph GT1M mean counts per minute was 0.30(*p *= 0.089). When comparing the total PASE score with the IPAQ total MET-minutes per week the correlation coefficient was 0.61 (*p *= 0.001).

**Table 3:** Construct validity of the total PASE score, and the scores for light, moderate and vigorous PA intensity

	Mean (SD)	Total score	Score for Light PA intensity	Score for Moderate PA intensity	Score for Vigorous PA intensity
Actigraph GT1M					

Average counts per minute, counts/min	370 (199))	**0.30**			

Total minutes in interval counts 0-99, min	4015 (736)				

Total minutes in interval counts 100-2019, min	1989 (669)		**0.20**	0.21	0.21

Total minutes in interval counts 2020-5999, min	294 (194)		0.46**	0.38*	0.11

Total minutes in interval counts > 6000, min	25 (50)		0.20	0.08	**0.29**

IPAQ					

Total IPAQ, MET-min/week	3476 (3609)	0.61**			

Walking MET-min/week	2098 (3145)		**0.31**	0.58**	0.05

Moderate Intensity, MET-min/week	707 (678)		0.20	**0.29**	0.16

Vigorous Intensity, MET-min/week	526 (869)		0.02	0.06	0.75**

*PA *physical activity, *PASE *Physical Activity Scale for the Elderly, *IPAQ *International Physical Activity Questionnaire, The categories of PASE and Actigraph GT1M and of PASE and IPAQ with the highest expected correlation coefficients are highlighted by bold text

*: significant at 0.05-level; **: significant at 0.01-level

For the different PA intensity items of the PASE we expected higher correlation coefficients with the respective categories of the Actigraph and the IPAQ. These comparisons are highlighted in Table 3. The correlation coefficients ranged from 0.10 to 0.35 between PASE and the Actigraph for the comparisons with the expected highest correlation, with only the correlation between the PASE item for moderate PA intensity and the respective Actigraph category reaching statistical significance. The correlation coefficients ranged from 0.29 to 0.75 between PASE and IPAQ for the comparisons with the expected highest correlation. Of these, the correlation between the PASE score for moderate PA intensity and the IPAQ score for walking, and the PASE score for vigorous PA intensity and the IPAQ score for vigorous PA intensity reached statistical significance.

## Discussion

This is the first study to address the test-retest reliability and the construct validity of the PASE in patients with hip OA, and the first study to evaluate the validity of the Norwegian version of the PASE. It is also one of relatively few studies evaluating the construct validity of a self-administered instrument for assessing PA by comparing it to an accelerometer, a method for direct measurement of PA, in patients with OA[13].

In our study we found that 67% of patients with hip OA fulfilled the recommendations of achieving at least 30 minutes of accumulated MVPA per day, but only 30% fulfilled the recommendations of achieving at least 30 minutes of MVPA per day in blocks of minimum 10 minutes. However, a larger percentage of the hip OA patients did fulfill the recommendations compared to the general Norwegian population. Only 20% of the general adult Norwegian population fulfill these recommendations, and a decline in the amount of PA was present after the age of 64 years. Mean counts per minute was 338, compared to 370 in our study[28]. The patients in our study were found to have high levels of PA when compared to other studies investigating levels of PA by accelerometers in OA patients[4,5,32]. Hirata et al.[32] found that women with hip OA were engaged in MVPA for 17 minutes per day, and only 14% met the recommendations of more than 30 minutes accumulated MVPA per day[32]. For patients with knee OA mean time spent on MVPA was 14-25 minutes per day[4,5] and 30% met the recommendations[4]. However, studies on PA levels in patients with knee OA may not be a valid comparison for the patients in our study. These previous studies[4,5,32] may have included patients with more progressive and severe OA than we did in our study, where patients with a Harris Hip Score below 60 points were excluded from participation. It is also important to stress that the hip OA patients in our study originally participated in a RCT where the importance of PA was emphasized through a patient education program, and this may have altered their PA levels. However, no changes in total PASE score was found for the 16 months follow-up of the RCT[20]. In addition, the possibility for selection bias is present, i.e. patients with a more positive attitude to PA might have been more likely to participate, and the education level was high. Thirty-nine percent of the patients in our study had more than 12 years of education, compared to 28% in the general Norwegian population (http://www.ssb.no/utniv). The levels of PA found in this study may therefore not be representative for the hip OA population in general.

PA has also been estimated in a representative sample of elderly Norwegians using PASE to assess physical activity[26]. The mean total PASE score was 127, quite consistent with the findings in our study on hip OA patients, where total PASE score was 143 and 125 at test and retest, respectively.

Measurement properties of an instrument are related to the population and context in which it is being used. In this study we evaluated the test-retest reliability of the PASE in patients with hip OA by calculating the ICC_2.1_, and in addition estimating the standard error of measurement (SEM) and the minimal detectable change (MDC). There are no absolute consensus regarding limits for what should be considered an acceptable ICC value. When instruments for assessing PA is evaluated, Terwee et al.[13,15] and Forsèn et al.[33] have suggested, and used, 0.70 as a cut-off for acceptable test-retest reliability. Based on this the test-retest reliability for the total PASE score was considered to be acceptable, with an ICC_2.1 _of 0.77. However, Terwee et al.[34] also suggested that the lower limit of the 96% CI of the ICC should exceed 0.60, and for the total PASE score the lower 95% CI was slightly lower than this, 0.56. The Norwegian version of PASE has previously been found to have acceptable reliability when tested in the general, elderly population, with an internal consistency of items (Cronbach's alpha) of 0.73, and test-retest reliability coefficient (Pearson's) of 0.93-0.99[26].

The SEM and MDC of the total PASE score were 31 and 87, respectively, indicating that 87 represents the smallest within-person change in score that can be interpreted as a real change, exceeding measurement error. However, a change exceeding the measurement error is not necessarily clinically relevant, which can be evaluated by estimating the Minimal Clinically Important Difference (MCID). It is advised that the MCID is estimated by using an anchor-based approach [35-37]. However, distribution-based approaches for estimating the MCID are also proposed, and the MCID has been found to equal approximately 0.5 SD at baseline[38] or approximately one SEM[39]. To be able to distinguish important changes from measurement error and to measure changes over time, the MCID should exceed the MDC[15], but by the smallest possible limit. The LoA indicates that if a subject completes a questionnaire twice, the second score could be as much as these limits smaller or larger than the first score, due to measurement error. Thus, the MCID should also lie outside the LoA[15]. Despite an acceptable test-retest ICC of the total PASE score, we consider the reliability to be moderate, due to large measurement error and wide LoA when compared to the mean total PASE score.

In our study, a significant decline in total PASE score of 18 points was present from test to retest, indicating a systematic error. We may therefore question whether the situation or the subjects actually were stable. When systematic error is present, this is often believed to occur due to a learning effect. However, this is not likely to be the case when the instrument of interest is a self-administered questionnaire. A more plausible explanation may be that wearing the Actigraph GT1M encouraged the patients to increase their activity levels, during the week the PASE referred to. According to Reiser and Schlenk[40] direct observations of PA by accelerometry may modify the pattern and level of PA among the participants, and may therefore bias the results.

Furthermore, this study evaluated the construct validity of the PASE by comparing it to an accelerometer, the Actigraph GT1M, and with another PA questionnaire, the IPAQ. As proposed by Terwee et al.[30] we tested predefined specific hypotheses including the expected direction and magnitude of correlations. In this study we found no significant correlation between the total PASE score and the Actigraph GT1M mean total counts per minute. The correlation coefficient was 0.30, in line with our a priori hypothesis. It was comparable to previous studies investigating the correlation between PASE and accelerometers in different populations, where correlations between 0.16-0.52 have been reported[17-19,41,42]. The correlation did not reach the cut-off for what we considered satisfactory correlation, above 0.50, as suggested by Terwee et al.[15]. Whereas self-reporting PA questionnaires is found to over-report levels of PA compared to accerelometers[43,44], Leenders et al.[45] found that accelerometers significantly underestimated PA related energy expenditure when compared to the doubly labelled water method. This may be due to some of its limitations. Accelerometers can of course only provide measurements for the particular time it is observed and recorded, cannot measure water exercises, and also fails to measure activities such as cycling and upper limb exercise correctly. Overestimation of total PA levels when using questionnaires and underestimation when using accelerometers, may to some degree explain the discrepancy between the two methods for measuring PA.

The correlation between total PASE score and IPAQ MET-minutes per week was moderate, with a correlation of 0.61, and barely within our a priori hypothesize of correlation between 0.6 and 0.9. Both PASE and IPAQ are self-administered with a seven day recall period, but household- and work activities is included in the PASE and weighed quite highly, whereas the IPAQ mainly captures leisure-time PA. This may, at least partly, explain the discrepancy between the two questionnaires. Both questionnaires were originally developed for use in a general population (generic), with PASE being specifically designed for an elderly population.

The PASE is not designed to be used to measure and report different PA intensity levels separately. One might therefore argue that acceptable test-retest reliability for the overall score is what is important. However, assessment of intensity seems valuable when investigating the effect of exercise and PA, especially for evaluating the dose-response relationship and to establish recommendations for patients with OA regarding amount and intensity. We therefore wanted to evaluate these specific items, to evaluate whether a PA questionnaire is able to provide reliable and valid data for PA intensity. The ICC_2.1 _for the sub-scores for household/work-related PA and for leisure-time PA was 0.69 and 0.53, respectively, and the ICC_2.1 _for the items for light, moderate and vigorous PA intensity was 0.46, 0.20 and 0.68, respectively. None of the ICC's for the sub-scores or the single item scores exceeded 0.7, which we interpreted as a cut-off for acceptable reliability, and the 95% CI were wide for all the sub-scores and items. The SEM and the MDC were also large compared to the mean values of the sub-scores and items, indicating moderate to low reliability.

Our a priori hypothesis; that the respective intensity categories of the PASE would correlate strongest with the respective intensity categories of the Actigraph GT1M, was confirmed for moderate PA intensity and vigorous PA intensity, but not for light PA intensity. However, all correlation coefficients were below 0.46. This indicated that the intensity items of the PASE were not able to distinguish between light, moderate and vigorous PA intensity, and we therefore consider the PASE not to be valid or reliable for assessing PA intensity. The item for moderate PA intensity of PASE correlated stronger with the IPAQ category for walking than the IPAQ category for moderate PA intensity. This may be due to the fact that the IPAQ includes a specific item for assessing walking activities, whereas walking activities are included in the items for light, moderate and vigorous PA intensity in the Norwegian version of PASE. Walking is a widespread leisure time activity in Norway, and is likely to be scored in the item for moderate PA intensity of the PASE, giving a higher correlation with the IPAQ walking compared to the IPAQ moderate PA intensity.

This study has some limitations. Both analysis of test-retest reliability and construct validity by comparing PASE to the Actigraph GT1M were based on data obtained from 33 patients. After referring a statistician, and based on that other studies have used similar sample sizes[19,33], we decided to include 40 patients in this study. According to the statistician a sample size between 30 and 40 is usually sufficient when evaluating outcome measurements that uses a continuous scale. According to Terwee et al.[15] sample size in reliability and/or validity studies evaluating PA assessment tools should exceed 50. A recently developed scoring system for rating methodological quality of measurement properties suggests that a sample size of 100 should be considered excellent, 50 as good, 30 as fair and under 30 as poor[46]. Correlation between PASE and IPAQ was only based on data from 25 patients. The Norwegian version of IPAQ has been validated for the Norwegian population, but has included an item "don't know" as an option for duration of activity which challenge the interpretation and the score calculations.

The use of Actigraph GT1M and the IPAQ to evaluate construct validity have some weaknesses. The doubly labeled water method is often considered to be the gold standard for measuring PA[15], but is seldom used to evaluate validity of PA questionnaires, as it is expensive, time-consuming and relies on access to both technical expertise and equipment. Only two studies have validated the PASE by comparing it to doubly labelled water, and found correlation coefficients of 0.28[47] and 0.68[48]. However, the doubly labelled water method is affected by the basal metabolic rate, and it cannot capture frequency, duration and intensity of activity. Accelerometers may therefore represent a more appropriate comparator because it can provide information on amount, pattern and intensity of PA, and therefore seem to measure the same construct as most PA questionnaires[15]. There is evidence for reasonable correlation between waist-worn accelerometers and the doubly labelled water method in adults, with correlations ranging from 0.30-0.83[49]. IPAQ was also included as a comparator because it is a widely used PA questionnaire, but like other questionnaires it is vulnerable to recall and reporting bias. Previous studies comparing IPAQ and accelerometers/activity monitors have reported correlation coefficients between 0.29 to 0.35[50-52]. However, Ainsworth[53] states that questionnaires may be suitable for assessing PA for most patients. More sophisticated methods, like accelerometers, provide more precise measurements, but are less practical for use in clinical settings. Kayes and McPherson[54] emphasize that PA questionnaires and accelerometers both have weaknesses, but that both methods are likely to assess important aspects of the PA construct. Use of both tools may therefore be appropriate to capture all aspects of PA.

## Conclusions

The test-retest reliability of the total PASE score in patients with hip OA was found to be moderate, based on an acceptable ICC_2.1_, but the large SEM, SDC and LoA indicate large measurement errors. The construct validity of the total PASE score was found to be poor when compared to the Actigraph GT1M accelerometer.

These findings suggest that PASE is not sufficient for assessing PA levels and intensity in patients with hip OA. Accelerometers provide a more precise tool of assessing amount and intensity of PA, and should preferably be included if feasible in studies where these dimensions are considered important.

## Competing interests

The authors declare that they have no competing interests.

## Authors' contributions

All authors participated in the design of the study, contributed in drafting the article, and read and approved the final manuscript. IS carried out the patient inclusion, handled the administration of questionnaires and accelerometers, and carried out the statistical analysis. EK carried out the processing of the Actigraph GT1M data.

## Pre-publication history

The pre-publication history for this paper can be accessed here:

http://www.biomedcentral.com/1471-2474/13/26/prepub
